# Supplementary material for: Adverse events associated with cyclophosphamide: A pharmacovigilance study using the FDA adverse event reporting system
Source: PLoS One. 2026 Jan 6;21(1):e0339757. doi: 10.1371/journal.pone.0339757 (PMC12774374; doi:10.1371/journal.pone.0339757)

PLOS ONE

Adverse events associated with cyclophosphamide: a pharmacovigilance study using the FDA adverse event reporting system

--Manuscript Draft--

**Manuscript Number:**	PONE-D-25-28004
**Article Type:**	Research Article
**Full Title:**	Adverse events associated with cyclophosphamide: a pharmacovigilance study using the FDA adverse event reporting system
**Short Title:**	a pharmacovigilance study using the FDA adverse event reporting system
**Corresponding Author:**	Yujuan Fu<br>Changchun University of Chinese Medicine<br>Changchun, CHINA
**Keywords:**	CHOP; FAERS; Adverse Reactions
**Abstract:**	Objective<br><br>Cyclophosphamide(CHOP) is a widely used chemotherapeutic agent belonging to the class of alkylating agents. It primarily inhibits the proliferation of tumor cells by interfering with DNA replication and transcription. CHOP has been approved for the treatment of various types of malignant tumors. The aim of this research is to assess adverse events linked to CHOP using real-world data.<br><br>Methods<br><br>This research collected and analyzed adverse event reports associated with CHOP from the FAERS(US Food and Drug Administration Adverse Event Reporting System) database spanning from the first quarter of 2004 to the third quarter of 2024. This study leveraged the FAERS database, extracting reports submitted exclusively by healthcare professionals where CHOP was designated as the primary suspect (PS). Four risk signal detection methods were employed: Reporting Odds Ratio (ROR), Proportional Reporting Ratio (PRR), Bayesian Confidence Propagation Neural Network (BCPNN), and Multiitem Gamma Poisson Shrinker (MGPS), to conduct a comprehensive analysis of CHOP-related ADRs.<br><br>Results<br><br>A total of 3,625 adverse event reports with CHOP as the primary suspect drug were obtained. Across 25 system organ classes (SOCs), four positive Preferred Terms(PTs) signals were detected. The most significant signal in SOCs was "blood and lymphatic system disorders" (ROR=6.64, 95% CI 6.49-6.79), while the most significant PTs signal was "high grade B-cell lymphoma Burkitt-like lymphoma recurrent" (ROR=613.63, 95% CI 123.85-3040.40).<br><br>Conclusion<br><br>This study provides compelling evidence of the presence of CHOP seven unexpected off-label AEs were also observed, such as mucoepidermoid carcinoma, hypotelorism of orbit, breakthrough COVID-19, airway remodeling, meningitis enteroviral, acute graft versus host disease, and gastrosplenic fistula. Additionally, the combination of CHOP and radiotherapy increased the risk of several severe AEs, such as hemorrhagic cystitis. These findings highlight the importance of caution when prescribing CHOP to high-risk individuals with a history of meningitis, cystitis or airway obstruction.
**Order of Authors:**	Hui Zhang
	Minghao Lin
	Dexi Zhao
	Yujuan Fu

Powered by Editorial Manager® and ProduXion Manager® from Aries Systems Corporation

Additional Information:	
Question	Response
**Financial Disclosure**<br><br>Enter a financial disclosure statement that describes the sources of funding for the work included in this submission. Review the submission guidelines for detailed requirements. View published research articles from *PLOS ONE* for specific examples.<br><br>This statement is required for submission and **will appear in the published article** if the submission is accepted. Please make sure it is accurate.<br><br>**Funded studies**<br>Enter a statement with the following details:<br>• Initials of the authors who received each award<br>• Grant numbers awarded to each author<br>• The full name of each funder<br>• URL of each funder website<br>• Did the sponsors or funders play any role in the study design, data collection and analysis, decision to publish, or preparation of the manuscript?<br><br>Did you receive funding for this work?	Yes
Please add funding details.<br>as follow-up to "**Financial Disclosure**<br><br>Enter a financial disclosure statement that describes the sources of funding for the work included in this submission. Review the submission guidelines for detailed requirements. View published research articles from *PLOS ONE* for specific examples.<br><br>This statement is required for submission and **will appear in the published article** if the submission is accepted. Please make sure it is accurate.	This work was supported by the Jilin Provincial Science and Technology Department Project (YDZJ202401092ZYTS).

**Funded studies**<br>Enter a statement with the following details:<br>• Initials of the authors who received each award<br>• Grant numbers awarded to each author<br>• The full name of each funder<br>• URL of each funder website<br>• Did the sponsors or funders play any role in the study design, data collection and analysis, decision to publish, or preparation of the manuscript?<br><br>Did you receive funding for this work?"	
Please select the country of your main research funder (please select carefully as in some cases this is used in fee calculation).<br>as follow-up to "**Financial Disclosure**<br><br>Enter a financial disclosure statement that describes the sources of funding for the work included in this submission. Review the submission guidelines for detailed requirements. View published research articles from *PLOS ONE* for specific examples.<br><br>This statement is required for submission and **will appear in the published article** if the submission is accepted. Please make sure it is accurate.<br><br>**Funded studies**<br>Enter a statement with the following details:<br>• Initials of the authors who received each award<br>• Grant numbers awarded to each author<br>• The full name of each funder<br>• URL of each funder website<br>• Did the sponsors or funders play any role in the study design, data collection and analysis, decision to publish, or preparation of the manuscript?<br><br>Did you receive funding for this work?"	CHINA - CN
**Competing Interests**	The authors have declared that no competing interests exist.

Use the instructions below to enter a competing interest statement for this submission. On behalf of all authors, disclose any competing interests that could be perceived to bias this work—acknowledging all financial support and any other relevant financial or non-financial competing interests.<br><br>This statement is required for submission and **will appear in the published article** if the submission is accepted. Please make sure it is accurate and that any funding sources listed in your Funding Information later in the submission form are also declared in your Financial Disclosure statement.<br><br>View published research articles from *PLOS ONE* for specific examples.<br><br>**NO authors have competing interests**<br><br>Enter: *The authors have declared that no competing interests exist.*<br><br>**Authors with competing interests**<br><br>Enter competing interest details beginning with this statement:<br><br>*I have read the journal's policy and the authors of this manuscript have the following competing interests: [insert competing interests here]*<br><br>* typeset	
**Ethics Statement**<br><br>Enter an ethics statement for this submission. This statement is required if the study involved:<br><br>• Human participants<br>• Human specimens or tissue<br>• Vertebrate animals or cephalopods<br>• Vertebrate embryos or tissues	N/A

• Field research<br><br>Write "N/A" if the submission does not require an ethics statement.<br><br>General guidance is provided below. Consult the submission guidelines for detailed instructions. **Make sure that all information entered here is included in the Methods section of the manuscript.**	

**Format for specific study types**<br><br>**Human Subject Research (involving human participants and/or tissue)**<br>• Give the name of the institutional review board or ethics committee that approved the study<br>• Include the approval number and/or a statement indicating approval of this research<br>• Indicate the form of consent obtained (written/oral) or the reason that consent was not obtained (e.g. the data were analyzed anonymously)<br><br>**Animal Research (involving vertebrate animals, embryos or tissues)**<br>• Provide the name of the Institutional Animal Care and Use Committee (IACUC) or other relevant ethics board that reviewed the study protocol, and indicate whether they approved this research or granted a formal waiver of ethical approval<br>• Include an approval number if one was obtained<br>• If the study involved *non-human primates*, add *additional details* about animal welfare and steps taken to ameliorate suffering<br>• If anesthesia, euthanasia, or any kind of animal sacrifice is part of the study, include briefly which substances and/or methods were applied<br><br>**Field Research**<br><br>Include the following details if this study involves the collection of plant, animal, or other materials from a natural setting:<br>• Field permit number<br>• Name of the institution or relevant body that granted permission	
**Data Availability**<br><br>Authors are required to make all data underlying the findings described fully available, without restriction, and from the time of publication. PLOS allows rare exceptions to address legal and ethical concerns. See the PLOS Data Policy and FAQ for detailed information.	Yes - all data are fully available without restriction

A Data Availability Statement describing where the data can be found is required at submission. Your answers to this question constitute the Data Availability Statement and **will be published in the article**, if accepted.<br><br>**Important:** Stating 'data available on request from the author' is not sufficient. If your data are only available upon request, select 'No' for the first question and explain your exceptional situation in the text box.<br><br>Do the authors confirm that all data underlying the findings described in their manuscript are fully available without restriction?	
**Describe where the data may be found in full sentences. If you are copying our sample text, replace any instances of XXX with the appropriate details.**<br><br>• If the data are **held or will be held in a public repository**, include URLs, accession numbers or DOIs. If this information will only be available after acceptance, indicate this by ticking the box below. For example: *All XXX files are available from the XXX database (accession number(s) XXX, XXX.).*<br>• If the data are all contained **within the manuscript and/or Supporting Information files**, enter the following: *All relevant data are within the manuscript and its Supporting Information files.*<br>• If neither of these applies but you are able to provide **details of access elsewhere**, with or without limitations, please do so. For example:<br><br>*Data cannot be shared publicly because of [XXX]. Data are available from the XXX Institutional Data Access / Ethics Committee (contact via XXX) for researchers who meet the criteria for access to confidential data.*<br><br>*The data underlying the results presented in the study are available from (include the name of the third party*	All relevant data are within the manuscript and its Supporting Information files.

and contact information or URL).<br>• This text is appropriate if the data are owned by a third party and authors do not have permission to share the data.<br><br>* typeset	
Additional data availability information:	

Powered by Editorial Manager® and ProduXion Manager® from Aries Systems Corporation

Manuscript Click here to access/download;Manuscript;Manuscript.docx

Adverse events associated with cyclophosphamide: a pharmacovigilance study using the FDA adverse event reporting system

Hui Zhang[1], Minghao Lin[1], Dexi Zhao[1,2], Yujuan Fu[1],

1 Changchun University of Chinese Medicine, Changchun, China;

2 The Affiliated Hospital to Changchun University of Chinese Medicine, Changchun, China;

*Correspondence to: Yujuan Fu, fuyujuan1111@163.com

Abstract

Objective: Cyclophosphamide(CHOP) is a widely used chemotherapeutic agent belonging to the class of alkylating agents. It primarily inhibits the proliferation of tumor cells by interfering with DNA replication and transcription. CHOP has been approved for the treatment of various types of malignant tumors. The aim of this research is to assess adverse events linked to CHOP using real-world data.

Methods: This research collected and analyzed adverse event reports associated with CHOP from the FAERS(US Food and Drug Administration Adverse Event Reporting System) database spanning from the first quarter of 2004 to the third quarter of 2024. This study leveraged the FAERS database, extracting reports submitted exclusively by healthcare professionals where CHOP was designated as the primary suspect (PS). Four risk signal detection methods were employed: Reporting Odds Ratio (ROR), Proportional Reporting Ratio (PRR), Bayesian Confidence Propagation Neural Network (BCPNN), and Multiitem Gamma Poisson Shrinker (MGPS), to conduct a

comprehensive analysis of CHOP-related ADRs.

Results: A total of 3,625 adverse event reports with CHOP as the primary suspect drug were obtained. Across 25 system organ classes (SOCs), four positive Preferred Terms(PTs) signals were detected. The most significant signal in SOCs was "blood and lymphatic system disorders" (ROR=6.64, 95% CI 6.49-6.79), while the most significant PTs signal was "high grade B-cell lymphoma Burkitt-like lymphoma recurrent" (ROR=613.63, 95% CI 123.85-3040.40).

Conclusion: This study provides compelling evidence of the presence of CHOP seven unexpected off-label AEs were also observed, such as mucoepidermoid carcinoma, hypotelorism of orbit, breakthrough COVID-19, airway remodeling, meningitis enteroviral, acute graft versus host disease, and gastrosplenic fistula. Additionally, the combination of CHOP and radiotherapy increased the risk of several severe AEs, such as hemorrhagic cystitis. These findings highlight the importance of caution when prescribing CHOP to high-risk individuals with a history of meningitis, cystitis or airway obstruction.

Keywords: CHOP; FAERS; Adverse Reactions

Introduction

Cyclophosphamide (CHOP) is a widely used chemotherapeutic agent belonging to the nitrogen mustard alkylating agents. Since its introduction in the 1950s, CHOP has become one of the cornerstone drugs in the treatment of various malignant tumors and autoimmune diseases[1]. After being metabolized in the liver, it is converted into

cytotoxic active substances, which inhibit the proliferation of tumor cells by cross-linking with DNA, thereby achieving an anti-tumor effect[2]. The clinical application of CHOP is extensive, covering hematological malignancies (such as non-Hodgkin's lymphoma, multiple myeloma, leukemia, etc.) and solid tumors (such as breast cancer, ovarian cancer, small cell lung cancer, etc.). It is metabolized into active forms in the body through liver cytochrome P450 enzymes (such as CYP2B6, CYP3A4, etc.). The main metabolic processes include: 4-hydroxyCHOP: the main active metabolite of CHOP; aldophosphamide: 4-hydroxyCHOP further decomposes into aldophosphamide; phosphoramide mustard and acrolein: aldophosphamide decomposes into cytotoxic phosphoramide mustard and acrolein within cells[3]. DNA cross-linking disrupt the double helix structure of DNA, hindering DNA replication and transcription, thereby inhibiting the proliferation of tumor cells[4]. Cross-linking also triggers DNA damage repair mechanisms, and when repair fails, cells initiate the apoptsosis program. In addition to its immunosuppressive effects, CHOP is also used to treat autoimmune diseases such as systemic lupus erythematosus and rheumatoid arthritis[5]. Although CHOP has demonstrated significant efficacy in clinical practice, its use is also associated with certain side effects, such as myelosuppression, alopecia, nausea, and vomiting. Long-term use may also increase the risk of secondary tumors. Therefore, in recent years, research has gradually shifted towards improving the targeting of CHOP, reducing side effects, and overcoming tumor resistance[6]. The application of novel drug delivery systems, combination therapy strategies, and gene editing technologies has provided new directions for the optimized use of CHOP[7].

While CHOP has exhibited notable effectiveness and activity, it necessitates vigilant attention to safety and the surveillance of specific toxic reactions. The FAERS, which is prevalently employed for adverse event (AEs) signal detection studies, serves as a valuable tool for monitoring and assessing the post-marketing safety of medications. Compared with previous studies, we used FAERS data mining technology to retrospectively detect and analyze the adverse reaction signals of CHOP, and further expanded the time span of the study.

2 Materials and Methods

2.1 Data Source

All our data comes from the FAERS Database (https://fis.fda.gov/extensions/FPD-QDE-FAERS/FPD-QDE-FAERS.html) from the first quarter of 2004 to the third quarter of 2024. This system consists of adverse event and medication error reports voluntarily submitted to the FDA by healthcare professionals and consumers. The database comprises seven subsets: patient information (DEMO), drugs (DRUG), indications (INDI), outcomes (OUTC), adverse reactions (REAC), medication timing (THER), and reporting country (RPSR). These subsets are linked and analyzed through the personal safety report code (primaryid) field. The data processing and analysis software used in this study is R software. This observational analysis utilizes the FDA Adverse Event Reporting System (FAER[illegible] database, which is updated quarterly and includes self-reported data from healthcare professionals (doctors, pharmacists, healthcare specialists, and registered nurses) and non-healthcare professionals (consumers, lawyers, sales representatives, etc.). The

FAERS database has been widely used to identify potential adverse drug reactions. It includes unique identifiers, reporting dates, reporting country/region, primary reporter qualifications, patient demographic information (such as gender, age, and weight), suspected and concomitant drugs and their indications, ADR occurrence date, and ADR manifestations.

2.2 Data Cleaning

Since the FAERS database can be reported by different institutions and the public, there are multiple versions of duplicate cases and non-standardized data. Therefore, before analysis, it is necessary to remove duplicates using the primaryid and caseid in the seven files. The primaryid is the unique number for identifying FAERS reports and the main linking field in the data files, while the caseid is the number for identifying cases. Each caseid corresponds to one Individual Case Safety Report (ICSR). When an ICSR is submitted multiple times subsequently, its caseid remains unchanged, but the primaryid will generate a new, larger number.

2.3 Data Extraction and Standardization

Data from the FAERS Database from 2004Q1 to 2024Q3 were extracted using 'CHOP' as the search term, and duplicate reports from the same patient were removed. The collected data mainly included the following characteristics of the drug-related reports: patient age, patient gender, type of reporter, adverse event outcomes (death, life-threatening, hospitalization, disability, and/or other), medications, and indications. The data were standardized using the preferred terms (PTs) from version 26.1 of the Medical Dictionary for Regulatory Activities (MedDRA), and adverse events were

categorized into different systems using the system organ class (SOCs).

2.4 Data Statistical Analysis

Summary of FDA-approved CHOP, as is shown in **Table 1**. To reduce the probability of false positive signals, this study used four methods to evaluate the correlation between drugs and adverse reaction events: Reporting Odds Ratio (ROR), Proportional Reporting Ratio (PRR), Bayesian Confidence Propagation Neural Network (BCPNN), and Multiitem Gamma Poisson Shrinker (MGPS). The higher the signal value of the adverse event, the stronger the signal, indicating a stronger correlation between the drug and the target adverse event. The calculation formulas and evaluation criteria for the four methods are listed in **Table 2**. All data cleaning, statistical analysis, and data visualization were performed using R software (version 4.4.0).

Table 1. Summary of FDA-approved Cyclophosphamide

Generic name	Brand name	Approval date
Cyclophosphamide	CYTOXAN、ENDOXAN、LYOPHILIZED CYTOXAN	1959

Table 2. Four major Methods used for signal detection.

Method	Formula	Threshold
ROR	ROR=ad/bc<br>95%CI=$e^{\ln(ROR)\pm 1.96(1/a+1/b+1/c+1/d)^{\wedge}0.5}$	a ≥ 3<br>ROR ≥ 2<br>95%CI (lower limit) > 1
PRR	PRR=a(c+d)/c/(a+b)<br>χ^2=[(ad-bc)^2](a+b+c+d)/[(a+b)(c+d)(a+c)(b+d)]	a ≥ 3<br>PRR ≥ 2<br>95%CI (lower limit) > 1
BCPNN	IC=$\log_2$a(a+b+c+d)/(a+c)/(a+b)<br>95%CI=E(IC) ± 2V(IC)^0.5<br>r=(a+b+c+d)^2/(a+b+1)/(a+c+1)	IC025>0

	$E(IC)=\log_2 a(a+b+c+d)^2/(a+b+c+d+r)/(a+b)/(a+c)$	
	$V(IC)=1/\ln 2(b+c+d+r-1)/(a+1)/(a+b+c+d+r+1)+(2+b+c+2d)/(a+b+1)/(a+b+c+d+r+3)$	
	$IC025=E(IC)-2V(IC)^{0.5}$	
	$EBGM=a(a+b+c+d)/(a+c)/(a+b)$	
EBGM	$95\%CI=e^{\ln(EBGM)\pm 1.96(1/a+1/b+1/c+1/d)^{0.5}}$	EBGM05>2
	$EBGM05=e^{\ln(EBGM)-1.96(1/a+1/b+1/c+1/d)^{0.5}}$	

Formula: a, number of reports containing both the suspect drug and the suspect adverse drug reaction; b, number of reports containing the suspect adverse drug reaction with other medications (except the drug of interest); c, number of reports containing the suspect drug with other adverse drug reactions (except the event of interest); d, number of reports containing other medications and other adverse drug reactions. ROR, reporting odds ratio; CI, confidence interval; N, the number of co-occurrences; PRR, proportional reporting ratio; χ^2, chi-squared; BCPNN, Bayesian confidence propagation neural network; IC, information component; IC025, the lower limit of the 95% one-sided CI of the IC; EBGM, empirical Bayesian geometric mean; EBGM05, the lower 95% one-sided CI of EBGM.

3 Results

3.1 Descriptive Analysis

A total of 28,591 reports were obtained for CHOP, with 13,364 from males, 7,910 from females, and 7,317 unknown, as shown in **Figure 1**. The majority of patients were aged 41-65 years and above, accounting for 2,640 cases. The main reporting countries were the United States, France, China, Canada, and Italy, as shown in **Figure 2**. The majority of reports were submitted by Physicians, accounting for 38.5%. The most common outcome was other serious (45.34%), followed by hospitalization (28.55%). The year with the highest number of reports was 2014, as shown in **Figure 3**. Adverse reactions mainly occurred within <7 days and 7-28 days, as shown in **Figure 4**.

3.2 Disproportionality Analysis

After screening CHOP, we identified a total of 83,576 positive risk signals for adverse events (AEs), spanning across 25 SOCs, as detailed in **Table 3.** A comparison with the existing CHOP product label reveals that the primary affected organ systems are categorized based on SOCs classification. Notably, blood and lymphatic system disorders emerged as the most prominent category, with 729 positive risk signals specifically for blood-related AEs. Among these, high-grade B-cell lymphoma Burkitt-like lymphoma recurrent exhibited the strongest positive signal, with a relative risk (RR) of 613.53 [95% CI: 123.85, 3040.4]. According to the ranking by ROR, the main systems affected by CHOP are blood and lymphatic system disorders, neoplasms benign, malignant and unspecified (including cysts and polyps), infections and infestations, and hepatobiliary disorders. The high-intensity PTs are high grade B-cell lymphoma Burkitt-like lymphoma recurrent RR [613.53, 95% CI (123.85, 3040.40)], precursor T-lymphoblastic lymphoma/leukAEsmia recurrent RR [327.29, 95% CI (138.76, 771.98)], and T-cell lymphoma recurrent RR [232.22, 95% CI (125.54, 429.54)], as shown in **Figure 5**.

Table 3 Disproportionate analysis in top 4 SOC

soc_english	Case Reports	ROR(95% CI)	PRR(95% CI)	chisq	IC(IC025)	EBGM (EBGM05)
blood and lymphatic system disorders	8771	6.64(6.49, 6.79)	6.05(5.93, 6.17)	37234.38	2.58(2.55)	6(5.89)
neoplasms benign, malignant and unspecified (incl cysts and polyps)	5487	2.49(2.42, 2.56)	2.39(2.34, 2.44)	4550.42	1.25(1.22)	2.39(2.33)
infections and	9174	2.16(2.11,	2.03(1.99,	5070.0	1.02(0.9	2.03(1.9

infestations		2.21)	2.07)	7	9)	9)
hepatobiliary disorders	1588	2.04(1.94, 2.15)	2.02(1.94, 2.1)	825.18	1.01(0.94)	2.02(1.94)

4 Discussion

4.1 CHOP 20 Year Span Analysis

This study maintains a certain continuity with previous studies in terms of data sources and analysis methods, both being based on the FDA Adverse Event Reporting System (FAERS) database for drug safety signal mining[8]. However, compared with previous studies, this study has the following significant advantages: First, in terms of data time span, this study includes data from 2004Q1 to 2024Q3, a total of 20 years, which is a significant improvement over the 3-5 year data span commonly used in previous studies. This long-term data collection not only captures more rare adverse events but also allows for the observation of the dynamic changes in drug safety signals, providing a more comprehensive temporal dimension for drug safety evaluation. Second, in terms of data volume, the increase in sample size significantly enhances the power of statistical analysis, aiding in the discovery of more potential safety signals. Moreover, the long-term data can better control the interference of seasonal factors on the analysis results, enhancing the reliability of the study conclusions. Lastly, this long-term study design enables us to observe the safety evolution of newly marketed drugs and the potential risk changes brought by the repurposing of old drugs, providing a more comprehensive reference for clinical medication safety[9].

4.2 Basic Situation of CHOP-Related Adverse Event Reports

The study results show that the number of adverse event reports for CHOP is related to gender. Among the CHOP reports with known gender, there are more reports from females than males, suggesting that when CHOP is used in female patients, there should be a heightened vigilance for the occurrence of adverse events. In terms of report age, the age group most affected by adverse events is 41-65 years old. However, there are still 11,912 reports with unknown age in this study, which may have a certain impact on the results. The top 5 countries in terms of reporting numbers are the United States, France, China, Canada, and Italy, with the United States having the highest number of reports. This may be related to the country where the database developer is located, the time of market availability in various countries, the country of origin of the drug, and the awareness, dissemination, and emphasis on drug adverse reactions among people in different countries. However, there are still 1,318 reports with unknown reporting countries in this study, which may have a certain impact on the results.

4.3 CHOP-Related AEs Involvement in SOCs

CHOP reports adverse events involving 25 SOCs in the MedDRA classification, which may be related to the broad scope of its mechanism of action. Among them, blood and lymphatic system disorders, neoplasms benign, malignant and unspecified (including cysts and polyps), infections and infestations, and hepatobiliary disorders have a higher number of reports and stronger signals, consistent with the descriptions in the product insert. This confirms the credibility of the methods used in this study.

4.4 CHOP-Related Adverse Events Ranked by Report Number

Among the 2,671 PTs, the highest number of positive reactions was found for high grade B-cell lymphoma Burkitt-like lymphoma recurrent. Current research indicates that CHOP is largely consistent with the product insert for precursor T-lymphoblastic lymphoma/leukAEsmia recurrent, T-cell lymphoma recurrent, metastases to reproductive organ, therapy responder, and chronic lymphocytic leukAEsmia recurrent. This phenomenon may be related to the central therapeutic role of the drug in aggressive B-cell malignancies: as a component of classic chemotherapy regimens such as CHOP, cyclophosphamide exerts a significant cytotoxic effect on highly proliferative Burkitt-like lymphoma cells by alkylating and disrupting DNA structure. It is worth noting that the concentration of positive reactions observed in this study may also reflect the urgent clinical need for treatment regimens for recurrent/refractory Burkitt lymphoma.

4.5 CHOP and Other Suspected PTs Signals

The study results show that the PTs for blood and lymphatic system disorders caused by CHOP include lymphoid tissue hypoplasia and acquired von Willebrand's disease. This suggests that when using CHOP clinically, there should be vigilance for adverse reactions in the blood system[10]. Considering that the indications for CHOP overlap with certain PTs symptoms in the tumor system, hepatobiliary system diseases, blood system, and infections, and that changes in related examination indicators may be due to the disease being treated with CHOP itself, or due to the effects of CHOP or subsequent infections, clinical judgment should be exercised during application[11]. Therefore, during the treatment of hepatobiliary and central nervous system diseases,

clinicians should carefully identify whether adverse events are present[12]. Additionally, seven unexpected off-label AEs were observed, such as mucoepidermoid carcinoma, hypotelorism of orbit, breakthrough COVID-19, airway remodeling, meningitis enteroviral, acute graft versus host disease, and gastrosplenic fistula. Immune suppression related events (e.g., breakthrough COVID-19, enterovirus meningitis) may be related to the profound lymphocytopenia induced by cyclophosphamide; Abnormal tissue repair (e.g., airway remodeling, gastro-splenic fistula) may reflect the drug's interference with fibroblast function; Secondary malignancies (e.g., mucoepidermoid carcinoma) require long-term follow-up to determine whether they are related to the carcinogenic risk of alkylating agents. The FAERS data cannot establish causality, and the sample size of off-label AEs is limited. Future prospective studies are needed to verify their associations. It is recommended to improve pharmacogenomic testing (such as CYP2B6 metabolizing enzyme analysis) in clinical practice to optimize the individualized medication strategy for cyclophosphamide. Currently, there are no large-scale epidemiological studies directly linking cyclophosphamide to the development of mucoepidermoid carcinoma (MEC). However, case reports suggest that immunosuppressed patients (such as those using CHOP after transplantation) may have an increased risk of salivary gland tumors. MEC is commonly found in the salivary glands (such as the parotid gland), and the association between alkylating agent exposure and head and neck tumors needs further validation.

4.6 Mechanism of Action of CHOP

The mechanism of action involves inhibiting cell proliferation by blocking DNA synthesis and nonspecifically killing lymphocytes, thereby exerting immunosuppressive and anti-inflammatory effects[13]. CHOP has been used for over 50 years in the treatment of lupus nephritis (LN) and is the most commonly used first-line drug for severe LN[14]. CHOP undergoes primary bioactivation in the liver via the mixed-function microsomal oxidase system, resulting in the formation of active alkylating metabolites. These metabolites disrupt the growth of rapidly proliferating malignant cells that are susceptible to their effects [15]. The liver serves as the primary site for CHOP activation, with approximately 75% of the administered dose being activated by hepatic microsomal cytochrome P450 enzymes, including CYP2A6, 2B6, 3A4, 3A5, 2C9, 2C18, and 2C19. Among these, CYP2B6 demonstrates the highest 4-hydroxylase activity. The activation of CHOP leads to the formation of 4-hydroxyCHOP, which exists in equilibrium with its open-ring tautomer, aldophosphamide. Both 4-hydroxyCHOP and aldophosphamide can be oxidized by aldehyde dehydrogenase to produce inactive metabolites: 4-ketoCHOP and carboxyphosphamide, respectively. Aldophosphamide can also undergo β-elimination to yield active metabolites such as phosphoramide mustard, acrolein, and nitrogen mustard. This spontaneous transformation can be facilitated by albumin and other proteins. Numerous in vitro and in vivo genotoxicity studies have demonstrated that CHOP possesses mutagenic and clastogenic properties[16]. Some studies have indicated that patients who developed cardiotoxicity received doses ranging from 120 to 270 mg/kg body weight within 1 to 8 days [17]. Low doses (e.g., <2 g/m^2 per

administration): mainly characterized by myelosuppression, which is usually manageable. High doses or cumulative doses (e.g., >6.5 g/m^2 or total dose >30 g): vigilance is required for irreversible organ damage and long-term secondary cancers. When used clinically, the balance between efficacy and toxicity must be strictly weighed, and the treatment regimen should be adjusted according to the patient's condition.

4.7 High-Risk Signal Analysis

Zhang Xiwen[18] reviewed the reproductive toxicity of CHOP, finding that it has strong reproductive toxicity in both males and females. Yang Mingyue[19] reported a case of cardiotoxicity caused by conventional-dose CHOP in a child with leukemia. Literature[20] indicates that in addition to reproductive toxicity, CHOP can also lead to osteoporosis and intestinal mucosal damage, with conclusions mostly derived from animal experiments and lacking clinical data support. Currently, there are no published studies on the adverse reactions of CHOP based on big data. The high-frequency PTs results statistically analyzed in this chapter are consistent with the drug instructions, indicating the reliability of the study results. CHOP mainly causes adverse events such as febrile neutropenia, fever, death, nausea, and decreased white blood cell count, which are consistent with the reported results that CHOP often induces blood and lymphatic system diseases and gastrointestinal reactions [21-24].

This study still has certain limitations. First, the FAERS database is a spontaneous reporting system, and the data have issues such as underreporting, misreporting, and missing information, with diverse sources of information and

potential reporting bias. Second, some patients' drug ADRs may be caused by complications or comorbidities. In clinical treatment, CHOP is often used in combination with other drugs, and the causal relationship between drugs and ADEs still needs further clinical research and evaluation. Additionally, the data in this study mainly come from European and American countries. Due to differences in ethnicity and region, the results may have certain deviations in China.

5 Conclusions

This study, based on the FAERS database, utilized the ROR method and BCPNN method to mine signals of adverse events related to CHOP, providing new supplements to the relevant adverse reactions in its drug instructions with more real - world data. In future clinical applications, it is necessary to strengthen the management of the standardized clinical use of CHOP and the management of off - label drug use. Medical staff should work together to judge the changes in relevant laboratory indicators during clinical application. CHOP should be used with caution in patients with hematological tumors, infections, and hepatobiliary diseases. At the same time, vigilance should be maintained against relevant adverse reactions. Patients should be informed in advance of the possibility of adverse reactions such as myelosuppression, immunosuppression, and infections. The necessity of routine blood cell counts should be explained. Patients should be guided to monitor their body temperature regularly and report any fever immediately. Vigilance should be maintained against situations such as hematuria and respiratory tract infections. Women of childbearing age should avoid pregnancy during the medication period as it

can easily cause fetal malformations. Side effects such as nausea, vomiting, stomatitis, poor wound healing, amenorrhea, premature menopause, infertility, and alopecia may be related to the administration of CHOP. Prudent consideration should be given to the use of this drug. In case of adverse reactions, patients should report to the doctor in a timely manner for treatment.

Ethics approval

All data were from the public database, and no ethical approval was needed.

Data availability statement

Database is available on the FAERS official website (https://fis.fda.gov/extensions/FPD-QDEFAERS/FPD-QDE-FAERS. html)

Funding

This work was supported by the Jilin Provincial Science and Technology Department Project (YDZJ202401092ZYTS).

Declaration of competing interest

The authors have no conflicts of interest to declare.

Acknowledgement

Conceptualization: Dexi Zhao and Yujuan Fu; Data Management: Chaoqun Song and Minghao Lin; Situation analysis: Hui Zhang, Chaoqun Song and Dexi Zhao; Methodology: Hui Zhang, Minghao Lin and Chaoqun Song; Software: Hui Zhang and Chaoqun Song; Original writing manuscriPTs: Hui Zhang and Chaoqun Song; Writing review Editors: Hui Zhang, Dexi Zhao and Yujuan Fu.

Competing interests

None.

Patient and public involvement

Patients and the public were not involved in our study.

Reference

[1] Zhong H, Lai Y, Zhang R, Daoud A, Feng Q, Zhou J, Shang J. Low Dose CHOP Modulates Tumor Microenvironment by TGF-β Signaling Pathway. Int J Mol Sci. 2020 Jan 31;21(3):957. doi: 10.3390/ijms21030957. PMID: 32023984; PMCID: PMC7038197.

[2] Kim J, Chan JJ. CHOP in dermatology. Australas J Dermatol. 2017 Feb;58(1):5-17. doi: 10.1111/ajd.12406. Epub 2016 Jan 24. PMID: 26806212.

[3] Sánchez-Sierra N, Perez-Somarriba M, Vázquez-Gómez F, González-Vicent M, Cabrera-Martín MN, Lassaletta A. Intracranial desmoplastic small round cell tumor after childhood acute myeloid leukemia treated with metronomic oral CHOP. Pediatr Blood Cancer. 2022 Nov;69(11):e29687. doi: 10.1002/pbc.29687. Epub 2022 Mar 26. PMID: 35338767.

[4] Scheinberg P, Townsley D, Dumitriu B, Scheinberg P, Weinstein B, Daphtary M, Rios O, Wu CO, Young NS. Moderate-dose CHOP for severe aplastic anemia has significant toxicity and does not prevent relapse and clonal evolution. Blood. 2014 Oct 30;124(18):2820-3. doi: 10.1182/blood-2014-05-573642. Epub 2014 Sep 3. PMID: 25185712; PMCID: PMC4215312.

[5] Ahlmann M, Hempel G. The effect of CHOP on the immune system: implications for clinical cancer therapy. Cancer Chemother Pharmacol. 2016 Oct;78(4):661-71. doi: 10.1007/s00280-016-3152-1. Epub 2016 Sep 19. PMID: 27646791.

[6] Ayza MA, Zewdie KA, Tesfaye BA, Wondafrash DZ, Berhe AH. The Role of Antioxidants in Ameliorating CHOP-Induced Cardiotoxicity. Oxid Med Cell Longev. 2020 May 10;2020:4965171. doi: 10.1155/2020/4965171. PMID: 32454939; PMCID: PMC7238386.

[7] Liu L, Liu B, Guan G, Kang R, Dai Y, Tang D. CHOP-induced GPX4 degradation triggers parthanatos by activating AIFM1. Biochem Biophys Res Commun. 2022 May 28;606:68-74. doi: 10.1016/j.bbrc.2022.03.098. Epub 2022 Mar 21. PMID: 35339754.

[8] Li JJ, Chen L, Zhao Y, Yang XQ, Hu FB, Wang L. Data mining and safety analysis of traditional immunosuppressive drugs: a pharmacovigilance investigation based on the FAERS database. Expert Opin Drug Saf. 2024 Apr;23(4):513-525. doi: 10.1080/14740338.2024.2327503. Epub 2024 Mar 27. PMID: 38533933.

[9] Li D, Qin C, Wang H, Du D, Wang Y, Du Q, Liu S. Assessing the risk of tumor lysis syndrome associated with the use of antineoplastic agents: a real-world pharmacovigilance study based on the FDA Adverse Event Reporting System database. Ther Adv Drug Saf. 2024 Sep 6;15:20420986241274909. doi: 10.1177/20420986241274909. PMID: 39247253; PMCID: PMC11380128.

[10] Gu JH, Samarneh M. Urotoxicity of CHOP: A Comparison Across Neoplastic, Autoimmune, and Transplant Indications. Cureus. 2024 Dec 5;16(12):e75180. doi: 10.7759/cureus.75180. PMID: 39764309; PMCID: PMC11701789.

[11] Barberino RS, Silva RLS, Palheta Junior RC, Smitz JEJ, Matos MHT. Protective Effects of Antioxidants on CHOP-Induced Ovarian Toxicity. Biopreserv Biobank. 2023 Apr;21(2):121-141. doi: 10.1089/bio.2021.0159. Epub 2022 Jun 13. PMID: 35696235.

[12] Ayza MA, Zewdie KA, Yigzaw EF, Ayele SG, Tesfaye BA, Tafere GG, Abrha MG. Potential Protective Effects of Antioxidants against CHOP-Induced Nephrotoxicity. Int J Nephrol. 2022 Apr 16;2022:5096825. doi: 10.1155/2022/5096825. PMID: 35469319; PMCID: PMC9034963.

[13] Delahousse J, Molina L, Paci A. "CHOP and analogues; a matter of dose and schedule for dual anticancer activities". Cancer Lett. 2024 Aug 28;598:217119. doi: 10.1016/j.canlet.2024.217119. Epub 2024 Jul 17. PMID: 39002693.

[14] Almalag HM, Alasmari SS, Alrayes MH, Binhameed MA, Alsudairi RA, Alosaimi MM, Alnasser GA, Abuzaid RA, Khalil N, Abouzaid HH, Alarfaj AS. Incidence of hemorrhagic cystitis after CHOP therapy with or without mesna: A cohort study and comprehensive literature review. J Oncol Pharm Pract. 2021 Mar;27(2):340-349. doi: 10.1177/1078155220920690. Epub 2020 May 1. PMID: 32356687.

[15] Luong SN, Isaacs A, Liu Z, Sin FE, Giles I. A systematic review and meta-analysis of the gonadotoxic effects of CHOP and benefits of gonadotropin releasing hormone agonists (GnRHa) in women of child-bearing age with autoimmune rheumatic disease. Expert Rev Clin Immunol. 2020 Mar;16(3):321-333. doi: 10.1080/1744666X.2020.1724091. Epub 2020 Mar 16. PMID: 32005081.

[16] Zirak MR, Karimi G, Rahimian R, Jafarian AH, Hayes AW, Mehri S. Tropisetron ameliorates CHOP-induced hemorrhagic cystitis in rats. Eur J Pharmacol. 2020 Sep 15;883:173310. doi: 10.1016/j.ejphar.2020.173310. Epub 2020 Jul 1. PMID: 32619674.

[17] Von Bernuth G, Adam D, Hofstetter R, et al. Cyclophosphamide cardiotoxicity. Eur J Pediatr. 1980;134(1):87-90. doi:10.1007/BF00442410

[18] Zhang Xiwen, Li Lele, Jiang Shuxin, etc. Progress in the reproductive toxicity of CHOP [J]. China Research Hospital, 2018,5 (01): 27-32.

[19] Yang Yueming, Liu Ling. Cardiac toxicity caused by conventional dose CHOP in childhood leukAEsmia A case report [J]. Chinese integrated Traditional Chinese and Western medicine

pediatrics,2014,6(04):383-384.

[20] Liu Junye, Li Lele, Jiang Shuxin, etc. Clinical application of CHOP and its toxicity prevention and treatment [J]. Study at Jilin University of Medicine Newspaper, 2019,40 (06): 449-451.

[21] Gurcan M, Esatoglu S N, Hamuryudan Ve, et al. Long term follow-up of Behçet's syndrome patients treated with CHOP.[J] .Rheumatology (Oxford), 2020, 59: 2264-2271.

[22] Jamali J, Dayo A, Adeel A, et al. A survey on gastrointestinal adverse drug reactions of Doxorubicin and CHOP combination therapy.[J] .J Pak Med Assocs, 2018, 68: 926-928.

[23] Tamang R, Bharati L, Khatiwada A P, et al. Pattern of adverse drug reactions asSOCsiated with the use of anticancer drugs in an oncology-based hospital of nepal.[J] .JMA J, 2022, 5: 416-426.

[24] Abdi M, FadAEse M, Jourabchi A, Karimzadeh H, Kazemi T. CHOP-Induced Infertility and the Impact of Antioxidants. Am J Reprod Immunol. 2024 Dec;92(6):e70014. doi: 10.1111/aji.70014. PMID: 39625043.

Figure captions

Fig 1. Proportion of adverse drug outcomes

Fig 2. Reporter country

Fig 3. Distribution of adverse events due to CHOP reported annually

Fig 4. Time of adverse events due to CHOP reported annually

Fig 5. Bubble diagram of the PTs

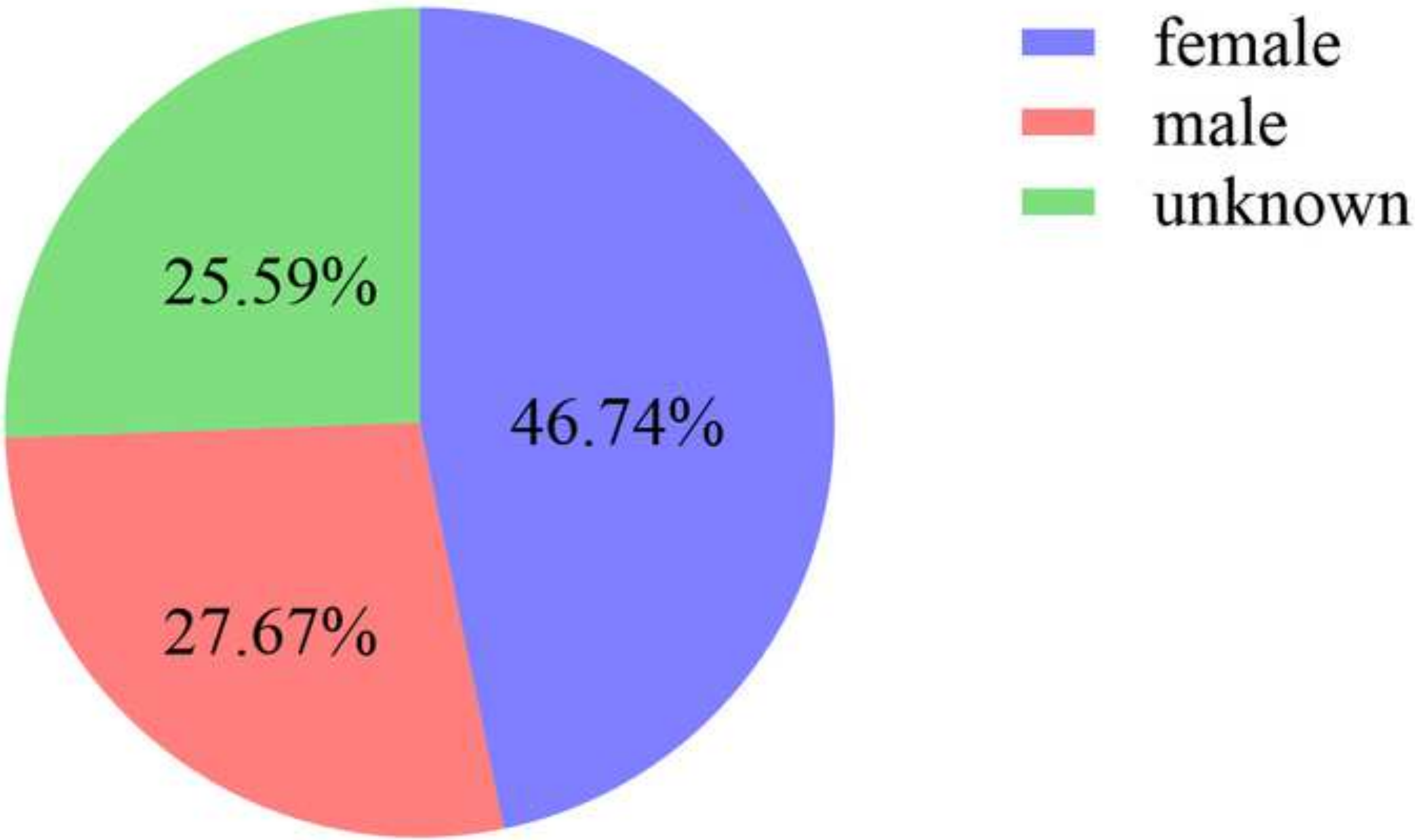
25.59%
46.74%
27.67%
female
male
unknown

Click here to access/download;Figure;Fig 2.tif

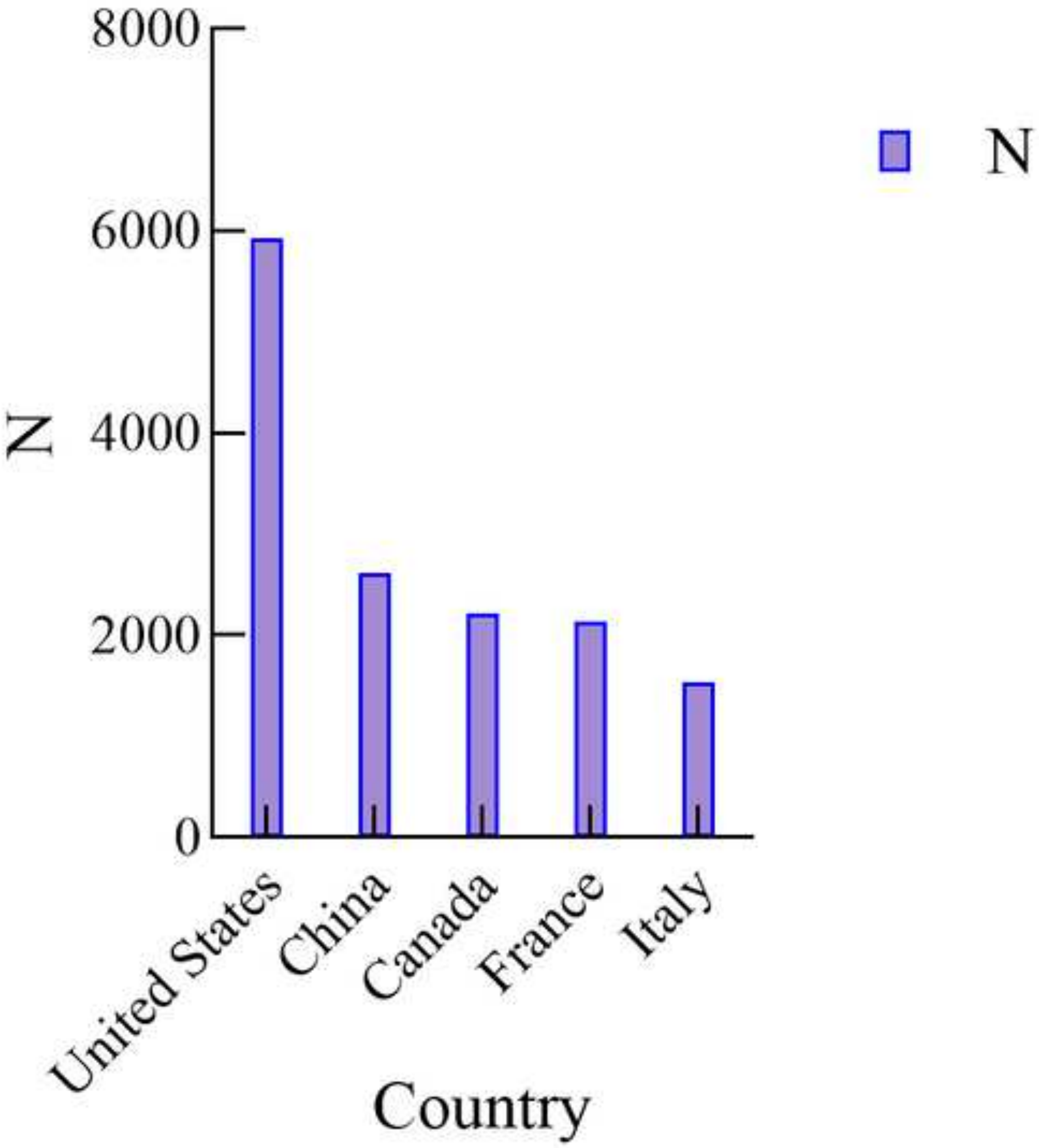

Figure

Click here to access/download;Figure;Fig 3.tif

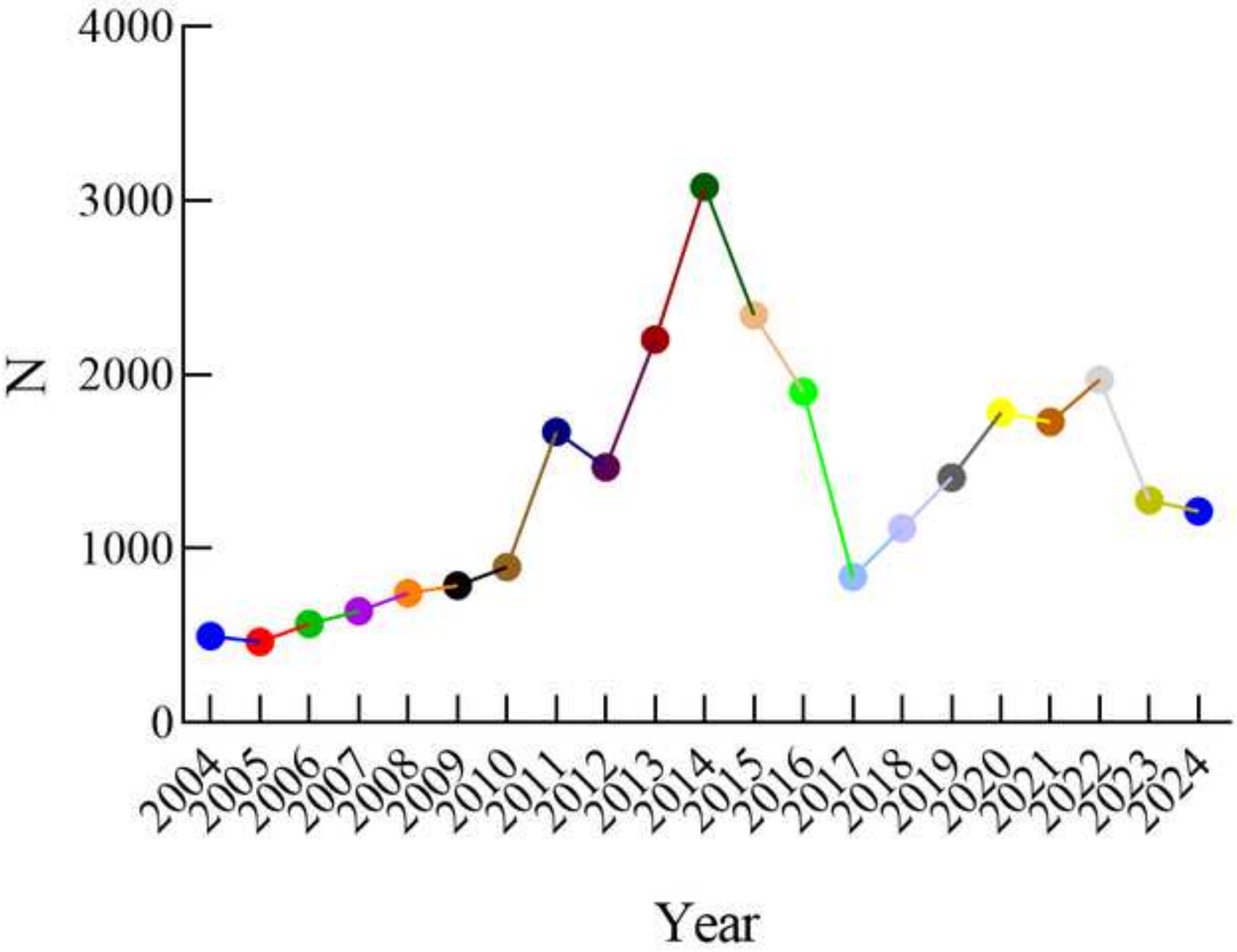

Figure

Click here to access/download;Figure;Fig 4.tif

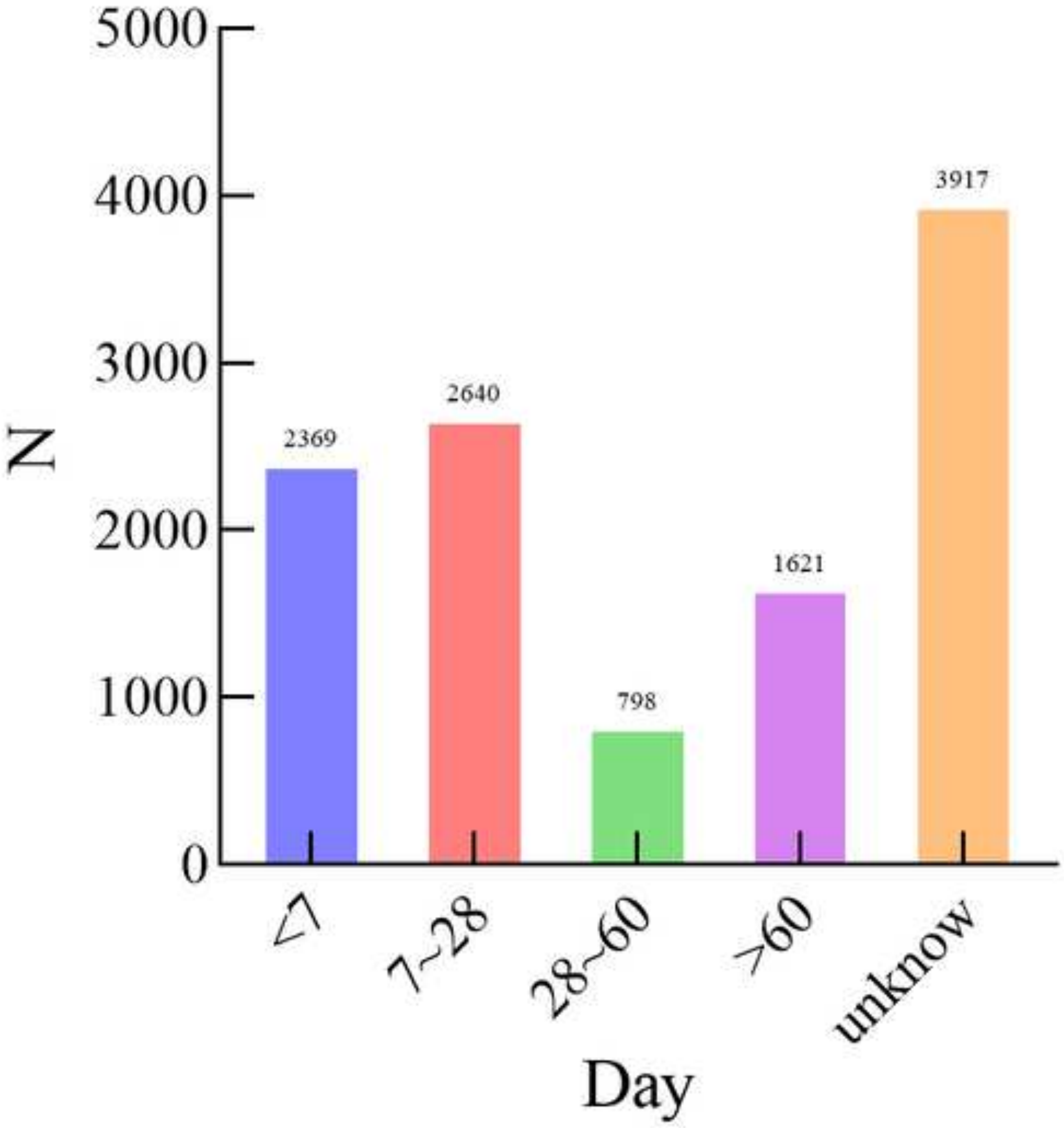

Click here to access/download;Figure;Fig 5.tif

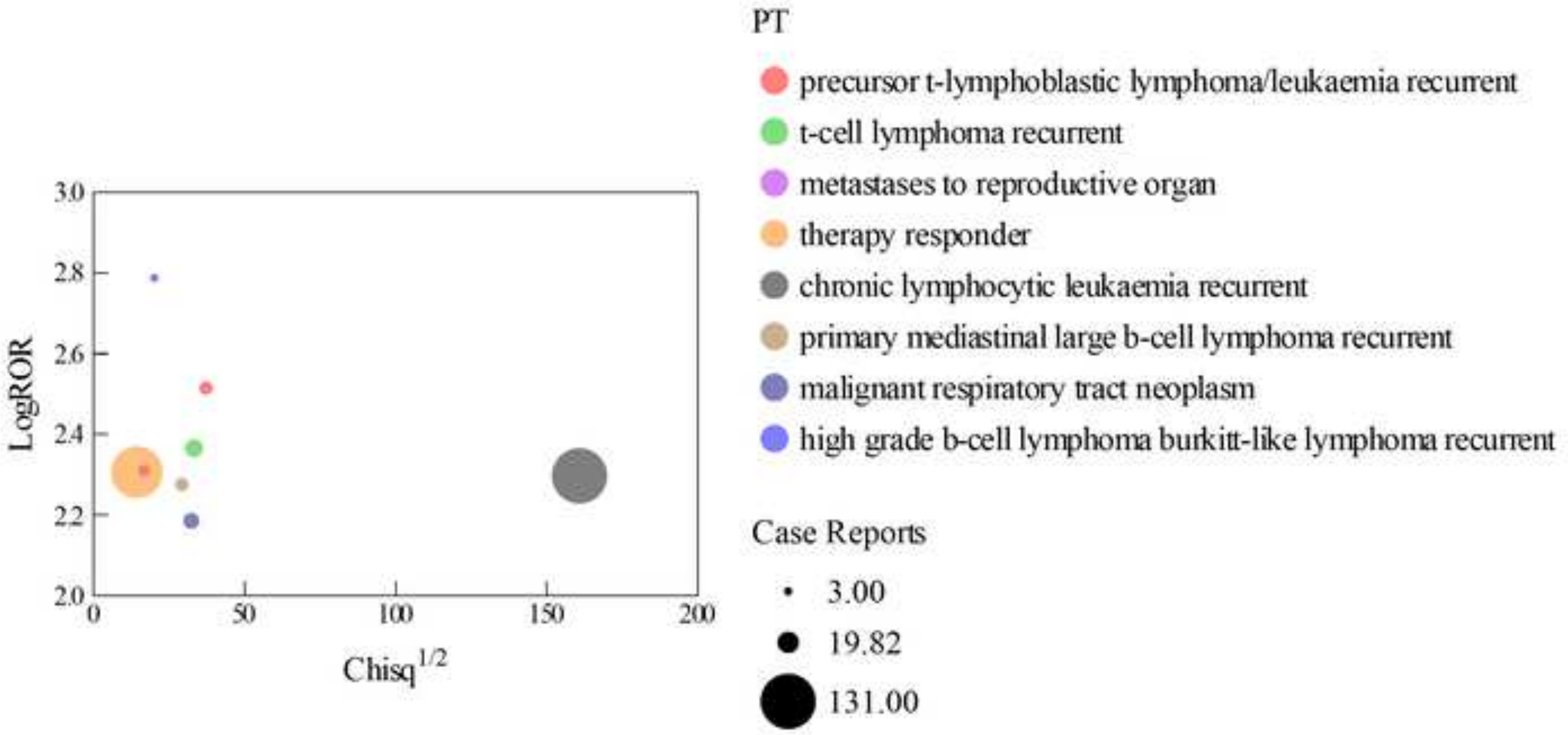

Supplement: Attachment — Submitted filename: PONE-D-25-28004.pdf [file pone.0339757.s003.pdf]
